# Complex patterns of genetic population structure in the mouthbrooding marine catfish, *Bagre marinus*, in the Gulf of Mexico and U.S. Atlantic

**DOI:** 10.1002/ece3.11514

**Published:** 2024-06-09

**Authors:** David S. Portnoy, Shannon J. O'Leary, Andrew T. Fields, Christopher M. Hollenbeck, R. Dean Grubbs, Cheston T. Peterson, Jayne M. Gardiner, Douglas H. Adams, Brett Falterman, J. Marcus Drymon, Jeremy M. Higgs, Erin L. Pulster, Tonya R. Wiley, Steven A. Murawski

**Affiliations:** ^1^ Marine Genomics Laboratory, Department of Life Sciences Texas A&M University – Corpus Christi Corpus Christi Texas USA; ^2^ Department of Biological Sciences Saint Anselm College Manchester New Hampshire USA; ^3^ Florida State University Coastal and Marine Laboratory St. Teresa Florida USA; ^4^ New College of Florida Sarasota Florida USA; ^5^ Florida Fish and Wildlife Conservation Commission Fish and Wildlife Research Institute, Indian River Field Lab Melbourne Florida USA; ^6^ Fisheries Research Support Mandeville Louisiana USA; ^7^ Mississippi State University Coastal Research and Extension Center Biloxi Mississippi USA; ^8^ Mississippi‐Alabama Sea Grant Consortium Ocean Springs Mississippi USA; ^9^ Center for Fisheries Research and Development The University of Southern Mississippi Ocean Springs Mississippi USA; ^10^ U.S. Geological Survey, Columbia Environmental Research Center Columbia Missouri USA; ^11^ College of Marine Science University of South Florida St. Petersburg Florida USA; ^12^ Havenworth Coastal Conservation Palmetto Florida USA

**Keywords:** ddRAD sequencing, directional selection, heterozygote advantage, larval dispersal, mouthbrooding

## Abstract

Patterns of genetic variation reflect interactions among microevolutionary forces that vary in strength with changing demography. Here, patterns of variation within and among samples of the mouthbrooding gafftopsail catfish (*Bagre marinus*, Family Ariidae) captured in the U.S. Atlantic and throughout the Gulf of Mexico were analyzed using genomics to generate neutral and non‐neutral SNP data sets. Because genomic resources are lacking for ariids, linkage disequilibrium network analysis was used to examine patterns of putatively adaptive variation. Finally, historical demographic parameters were estimated from site frequency spectra. The results show four differentiated groups, corresponding to the (1) U.S. Atlantic, and the (2) northeastern, (3) northwestern, and (4) southern Gulf of Mexico. The non‐neutral data presented two contrasting signals of structure, one due to increases in diversity moving west to east and north to south, and another to increased heterozygosity in the Atlantic. Demographic analysis suggested that recently reduced long‐term effective population size in the Atlantic is likely an important driver of patterns of genetic variation and is consistent with a known reduction in population size potentially due to an epizootic. Overall, patterns of genetic variation resemble that of other fishes that use the same estuarine habitats as nurseries, regardless of the presence/absence of a larval phase, supporting the idea that adult/juvenile behavior and habitat are important predictors of contemporary patterns of genetic structure.

## Abstract 2

Gafftopsail catfish show high levels of genetic structuring across their distribution in the U.S., Atlantic and Gulf of Mexico. Non‐neutral data present two contrasting signals of structure and historical demography is likely an important driver of patterns of neutral and non‐neutral genetic variation. Results of this study are similar to what is seen in other fishes that use the same estuarine habitats as nurseries, but gafftopsail catfish lack a larval phase, supporting the idea that adult/juvenile behavior and habitat use are important predictors of contemporary patterns of genetic structure.

## 1 INTRODUCTION

Contemporary patterns of genetic structure are the result of interactions among mutation, genetic drift, gene flow, and selection across many generations (Kidner et al., 2021). As such, the relative importance of these microevolutionary forces in shaping genetic diversity is temporally dynamic, changing with the demography of populations (Marko & Hart, 2011). Furthermore, for species distributed across heterogeneous environments, the relative importance of each force and the nature of their interactions may vary across space (Garant et al., 2007). Ultimately, it is allele frequency changes caused by these forces that underlie all evolutionary processes (Mayr & Provine, 1980), and those changes can be manifested at genome‐wide scales (e.g., drift interacting with demographic change; Hollenbeck et al., 2019) or as locus‐specific phenomena (e.g., selection operating at local scales in the genome; Gagnaire & Gaggiotti, 2016; Hoey & Pinsky, 2018, O'Leary et al., 2021), making genomic perspectives vital for assessments of genetic population structure (Bernatchez et al., 2017; Luikart et al., 2003).

For marine bony fishes, the proper interpretation of patterns of genetic structure is critical because this information is used by fisheries managers to delineate stock (i.e., population) boundaries and provide insight into both the long‐ and short‐term sustainability of management units (Hilborn et al., 2003). Marine systems tend to be open (i.e., lacking obvious physical boundaries) and most marine bony fishes have large population sizes and are highly dispersive either as adults or especially during prolonged larval phases, and as a result, the traditional paradigm was that species should show genetic, and hence, demographic homogeneity over large spatial expanses (Waples, 1998). More recent empirical and theoretical studies have demonstrated that genetic structuring in marine fishes can occur at much smaller scales than originally predicted (Hauser & Carvalho, 2008), but the processes that lead to genetic discontinuities in the absence of hard barriers remain an important topic of research.

Gafftopsail catfish are distributed in coastal shelf habitats and saline estuaries in the western North Atlantic off New England and southward along the East Coast of the United States (hereafter U.S.) and throughout the Gulf of Mexico (Marceniuk et al., 2022). The species exhibits a seasonal migratory pattern related to reproduction (Yáñez‐Arancibia & Lara‐Dominguez, 1988). In U.S. waters along the South Atlantic coastline (Atlantic) and the Gulf of Mexico (Gulf), spawning is thought to occur along the nearshore shelf in the late spring into summer, with females producing small clutches of large eggs that are fertilized and taken into the male's mouth (Muncy & Wingo, 1983). Fertilized eggs and developing fry are subsequently carried by the male for a period that may exceed 60 days and released as small, fully formed juveniles in estuarine habitat (Gudger, 1918). While gaff‐topsail catfish lack a dispersive larval phase, their use of estuaries as important habitat during the juvenile phase parallels what is seen in several co‐distributed marine fishes that do have dispersive larval phases (Able, 2005).

While studies on the biology of gafftopsail catfish and other ariids are limited, there appear to be several adaptations associated with mouthbrooding. For females, these include the production of some of the largest eggs of any bony fish, which are attached to nonfunctional hyaline eggs, and seasonal modification of pelvic fins thought to facilitate the transfer of eggs to the male's mouth (Eastman et al., 1970; Gunter, 1947; Merriman, 1940). For males, adaptations include a period of fasting during incubation and seasonal changes to the volume of the oral cavity (Gudger, 1918; Lee, 1937). In addition, limited tagging data suggest that adult gafftopsail catfish show interannual fidelity to estuarine habitat: 11 of 14 gafftopsail catfish tagged with acoustic transmitters in Apalachicola Bay, Florida, emigrated from the system then returned the next year, with most individuals returning for a third year (Peterson & Grubbs, Florida State University, oral communication, July 2023). The combination of extreme parental investment in offspring and fidelity to spatially heterogeneous habitats during a critical life stage suggests the strong potential for localized adaptation.

Despite the importance of gaff‐topsail catfish in regional fisheries and as a dominant midwater predator in nearshore ecosystems (Mendoza‐Carranza & Hernández‐Franyutti, 2005), they are understudied, and there have been only a few assessments of genetic population structure in this species and other ariid catfishes (Arroyo‐Zúñiga et al., 2021; Avise, 1992; Avise et al., 1987; Santos & Quilang, 2012). Therefore, to understand patterns of population structure, this study assessed locus‐specific and genome‐wide patterns of variation in samples collected across the species' range using nuclear‐encoded single nucleotide polymorphism (SNP)‐containing loci (microhaplotypes) to decouple drift process from natural selection. Because gaff‐topsail catfish are currently distributed in areas impacted by recent glacial cycles (Atlantic and Gulf, Portnoy et al., 2014), modeling based on site frequency spectra was used to understand the impact of historical demography on patterns of contemporary variation.

## 2 METHODS

### 2.1 Sampling and library prep

**FIGURE 1:**
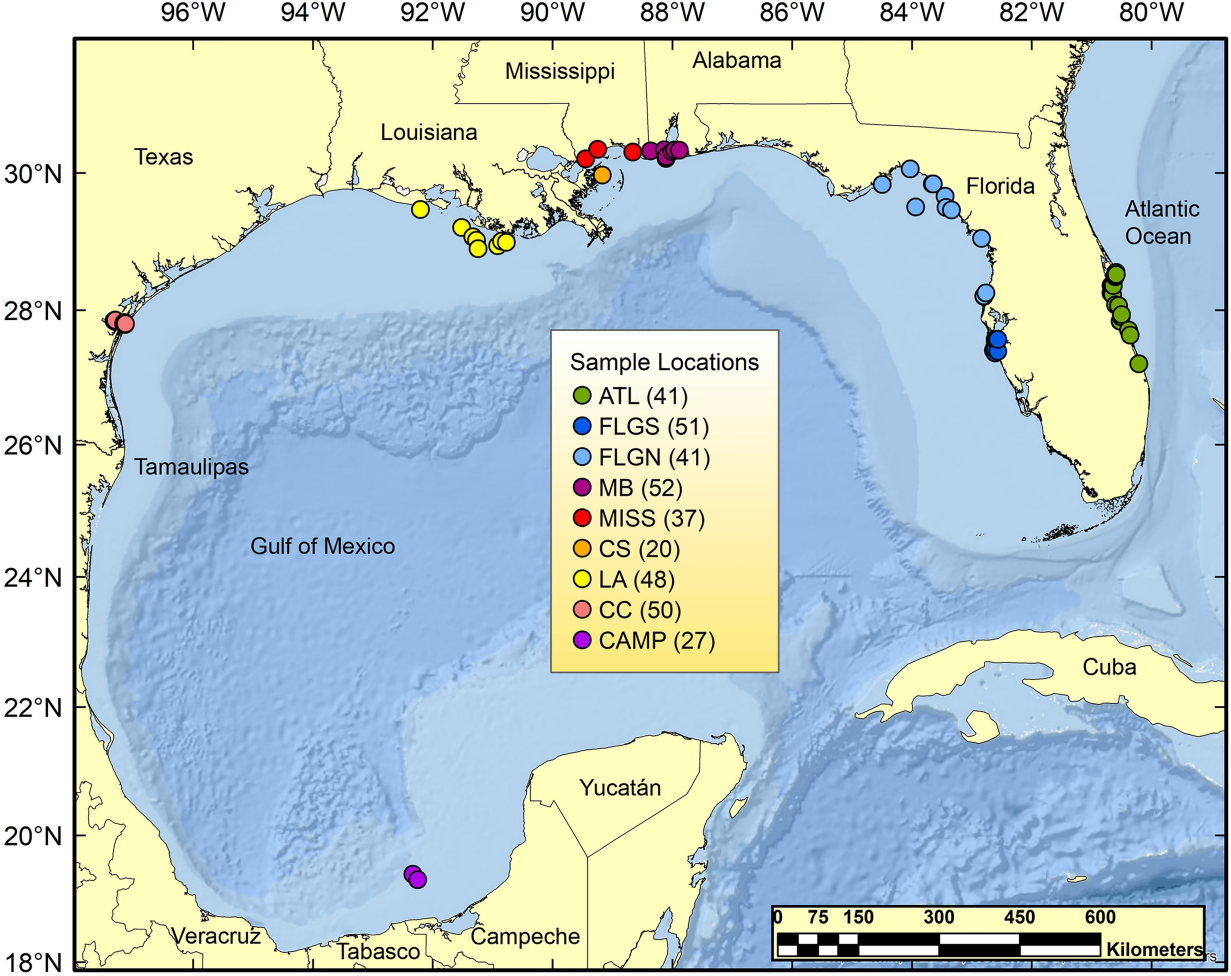
Map of the sampling distribution of gafftopsail catfish, *Bagre marinus*, in the U.S. South Atlantic and Gulf of Mexico. The nine geographic locations are Indian River Lagoon, Florida (ATL) in the Atlantic, Tampa Bay, Florida (FLGS), North of Tampa Bay, Florida (FLGN) Mobile Bay, Alabama (MB), Mississippi Sound, Mississippi (MISS), Chandeleur Sound, LA (CS), off Louisiana west of the Mississippi River (LA), Corpus Christi Bay, Texas (CC), and the Bay of Campeche, Mexico (CAMP). Numbers next to abbreviated names indicate sample sizes.

Fin clips were obtained from 382 mixed‐age samples of gafftopsail catfish collected from nine geographic sampling locations (hereafter locations; Figure 1) from 2015 to 2018: one in the Atlantic in the Indian River Lagoon, Florida, and adjacent coastal waters (ATL) and eight in the Gulf. Locations in the Gulf were near Tampa Bay, Florida (FLGS), North of Tampa Bay, Florida (FLGN), near Mobile Bay, Alabama, (MB), in Mississippi Sound, Mississippi (MISS), in Chandeleur Sound, LA (CS), off Louisiana west of the Mississippi River (LA), in Corpus Christi Bay, Texas (CC) and the Bay of Campeche, Mexico (CAMP). All locations were selected because they represent inshore habitats used by mouthbrooding males for parturition and by juveniles as nursery habitat, except CAMP which was opportunistically sampled further offshore. Sampling took place as part of surveys routinely conducted by state or academic entities, the latter following approved animal care protocols. All fin clips were preserved in 20% DMSO‐0.25M EDTA‐saturated NaCl buffer (Seutin et al., 1991) and stored at room temperature until the time of extraction.

DNA was extracted using Mag‐Bind Tissue DNA kits (Omega Bio‐Tek, Norcross, GA) and 500–1000 ng of high‐quality genomic DNA was used in a modified version of the ddRAD genomic library preparation method (Peterson et al., 2012). Briefly, genomic DNA was digested with two restriction endonucleases (*Eco*RI, *Msp*I), and a barcoded adapter was ligated to *Eco*RI sites while a common adapter was ligated to *Msp*I sites. Following adapter ligation, individuals were pooled by index and size‐selected using a Pippin Prep size‐selection system (Sage Science, Beverly, MA) to a standard size range (338–412 base pairs). Polymerase chain reaction (PCR) amplification of fragments was performed to incorporate adaptors necessary for annealing to an Illumina flow cell and index‐specific identifiers. Index pools were then combined into libraries of approximately 150 individuals spread across the geographic range of sampling and duplicate individuals (technical replicates), and three libraries were sequenced (150 bp paired‐end) each on a lane of an Illumina HiSeq 4000 DNA sequencer at GeneWiz®, New Jersey, USA.

### 2.2 Genotyping

RAD sequences retrieved from each run were demultiplexed using *process_radtags* (Catchen et al., 2011), and quality trimming, reduced‐representation reference assembly, read mapping, and SNP calling were performed using the *dDocent* pipeline (Puritz et al., 2014). The ten individuals with the highest number of reads were selected from each lane for de novo reduced‐representation reference assembly using the overlapping read (OL) assembly option in *dDocent*. Similarity threshold for clustering (*c* = 0.8), minimum within individual coverage (*K*1 = 5), and minimum number of individuals a read must occur in to be included (*K*2 = 2) were chosen after comparing mapping statistics for ten individuals randomly chosen from each library and mapped to references generated for *c* = 0.8, *K*1 = 2–10, and *K*2 = 1–10 using *BWA* (Li & Durbin, 2009) to maximize the number of reads mapped as a proper pair and minimize reads where forward and reverse reads mapped to different contigs. The constructed reduced‐representation reference encompassed a total of 10,874,990 base pairs across 37,872 fragments (mean 287 bp; mode 307 bp).

Reads were mapped to the reduced‐representation reference using *BWA* (Match = 1, mismatch penalty = 3 and gap penalty = 5; Li, 2013) and SNPs called using *freebayes* (Garrison & Marth, 2012). The resulting data set was filtered to remove low‐quality and artefactual SNPs, paralogs, and low‐quality individuals using *vcftools* (Danecek et al., 2011) and custom scripts following O'Leary et al. (2018), allowing for the retention of SNPs with more than two alleles. Genotypes with quality <20 and <5 reads were coded as missing, retaining loci with quality >20, genotype call rate >90%, and mean depth 15–300. Loci were also filtered based on allelic balance (remove SNPs <0.25 and >0.75), mapping quality ratios (remove SNPs <0.25 and >1.75), strand balance (remove SNPs with >100× more forward alternate reads than reverse alternate reads and >100× more forward reverse reads than reverse alternate reads), paired status, depth/quality ratio (<0.2), and excess heterozygosity (remove SNPs >0.5 and that deviate significantly from the expectations of Hardy–Weinberg Equilibrium). Individuals with >25% missing data were removed. Finally, *rad_haplotyper* (Willis et al., 2017) was used to merge SNPs on the same fragments into SNP‐containing loci (hereafter microhaplotypes), by using a random sample of 20 reads per locus and recording all possible haplotypes, and then discarding haplotypes that are not possible given the SNPs present in the final dataset. Loci are flagged as paralogs if too many haplotypes are called given SNP genotypes. Genotyping error is flagged if an individual has too few haplotypes given SNP genotypes. The resulting haplotyped data set was further filtered to remove loci haplotyped in <90% of individuals, flagged as potential paralogs in >4 individuals, or as affected by genotyping error in >10 individuals. Technical replicates were compared to assess genotyping error, and loci systematically affected by genotyping error or flagged as deviating significantly from the expectations of Hardy–Weinberg Equilibrium (HWE) in >5 locations were removed.

### 2.3 Test for homogeneity and patterns of variance

Homogeneity in allele and genotype distributions among locations was tested using a single‐level, locus‐by‐locus analysis of molecular variance (AMOVA) to account for uneven levels of missing data among loci (Weir & Cockerham, 1984). Analysis was implemented in arlequin *v*. 3.5 (Excoffier & Lischer, 2010) and significance determined at an *α*‐level of .05 by permuting individuals among locations 20,000 times and bootstrapping the data 20,000 times to generate 95% confidence intervals. To visualize differences among locations, principal components analysis (PCA) was performed using the R (R Core Team, 2022) package *adegenet v*.1.7‐18 (Jombart, 2008; Jombart & Ahmed, 2011).

### 2.4 Identification of neutral and non‐neutral loci

Two methods were explored to identify loci potentially under directional selection (outlier loci). First, the Bayesian modeling approach for outlier detection, implemented in bayescan (Foll & Gaggiotti, 2008), was run with 25 pilot runs of 5000 iterations, followed by a burn‐in of 50,000 iterations and 500,000 iterations sampled 10,000 times, and a *q*‐value of 0.05. Second, the Fdist method (Antao et al., 2008) for outlier detection implemented in arlequin was used with 20,000 coalescent simulations and a strict island model. Correction for multiple testing employed the Benjamini and Hochberg (1995) False Discovery Rate procedure (BH‐FDR). Because marine fishes typically have low background *F*_ST_ (Waples, 1998), assessments for loci under balancing selection were not conducted in either outlier analysis.

Because not all selection leads to patterns of differentiation consistent with assumptions of *F*_ST_ outlier analyses (Forester et al., 2018), redundancy analysis (RDA), implemented in *vegan* v. 2.5‐6 (Oskanen et al., 2022), was used to explore associations between components of genetic and environmental variation and identify environmentally associated loci (env loci). Two constraining matrices were created, one describing spatial relationships between individuals and the other describing aspects of environmental heterogeneity between discrete sampling locations. Spatial relationships were described using Moran's Eigenvector Maps (MEMs; Dray et al., 2006), with geographic distance calculated using the R‐package *codep* (Guenard et al., 2018), as the shortest distance between points on a sphere (great circle distance) using latitude and longitude data associated with the capture location of each individual. A final set of MEMs was then selected using stepwise forward selection, performed using the *ordiR2step* function in *vegan* with 999 permutations and *α*‐level of .05. An initial set of 68 environmental variables (Table S5) were obtained from the Bio‐Oracle (Assis et al., 2018; Tyberghein et al., 2012) and MARSPEC (Sbrocco & Barber, 2013) data sets, with the final set selected as above.

Significant factors (MEMs and environmental variables) were included in a full model, with significance determined at an *α* level of .01 using 1000 permutations. Then, variance partitioning was used to compare the relative contribution of geographic distance, environmental difference, and shared effects to observed patterns of genetic variation with the significance of each component determined at an *α*‐level of .01 using 1000 permutations. Finally, the environmental data were run separately to identify loci strongly associated with environmental variables by flagging alleles with a Mahalanobis distance ≥20.51 for *p* < .001 (df = 5).

The full dataset was then subdivided into “neutral” and “non‐neutral” datasets. The non‐neutral dataset was composed of outlier loci putatively under directional selection identified by at least one of the two detection methods and env loci. The neutral dataset contained all remaining loci. Each dataset was analyzed separately in all downstream analyses unless noted.

### 2.5 Genetic variation within and among locations

To identify the number of genetically distinct groups in the data and assess for patterns of hierarchical structure, a Discriminant Analysis of Principal Components (DAPC; Jombart et al., 2010) was used as implemented in *adegenet*, using *K*‐means clustering (*K* = 1–40) with the optimal number of clusters identified by comparing Akaike information criterion (AIC) values. To ensure sufficient variance was retained to discriminate among groups but not overfit the data, the optimum number of principal components to retain was determined using stratified cross‐validation. Missing data was imputed using mean allele frequencies. Based on the results, individuals were clustered for *K* = 2–5 (20–50 PCs retained) and membership probabilities of individuals to each inferred cluster were calculated. For selected *K* values, the percentages of variance explained by differences between groups and by differences between locations within groups were calculated in a locus‐by‐locus analysis of molecular variance (AMOVA) framework using arlequin. Pairwise *F*_ST_ was then estimated between each location using arlequin, with significance assessed at an *α*‐level of .05, by permutating individuals among locations 10,000 times. Correction for multiple testing employed BH‐FDR, as above.

Expected heterozygosity (*H*_e_; Nei, 1973) and rarified allelic richness (*A*_R_; El Mousadik & Petit, 1996) were estimated for each location using *hierfstat v*0.5‐10 (Goudet, 2005). Friedman's tests in R were used to test homogeneity among locations for each measure of diversity. Post hoc Wilcoxon signed‐rank tests in the R package *coin* v1.4‐3 (Hothorn et al., 2023) were used to assess for pairwise differences between locations. Correction for multiple testing employed BH‐FDR.

Chromosomal architecture has been implicated as an important driver in local adaptation because it can lead to co‐adapted loci that sort together and suppression of recombination that can reshuffle linked co‐adapted variation (Schwander et al., 2014). While some genomic resources are available for the order Siluriformes, none are appropriate for use in this study because the order arose at least 73 million years ago and contains more than 3000 named species and many undescribed species, spread across more than 30 families (Ferraris, 2007; Kappas et al., 2016; Sullivan et al., 2006), and available genomes are from taxa distant from the family Ariidae. Therefore, linkage disequilibrium network analysis (LDna; Kemppainen et al., 2015) was employed using the R package *LDna* v2.0 to identify single outlier clusters (SOCs), which are exclusive clusters of loci that show elevated linkage disequilibrium (LD) with one another relative to genome‐wide LD. The rationale behind this analysis is that SOCs represent sets of loci either responding to the same microevolutionary force (in this case directional selection), segregating together due to chromosomal architecture, or a combination of both. Linkage disequilibrium was estimated for all pairs of loci across the entire data set and across all locations as *r* ^2^, following the Burrows method (Weir, 1996) as implemented in nestimator *v*2.01 (Do et al., 2014) with a minimum allele frequency of 0.01. Subsequently, the analysis was used to generate a series of networks where the nodes were loci and threshold LD values were used to constrain cluster formation. The threshold was lowered iteratively allowing further clustering. By measuring the change in median LD as clusters became more inclusive (*λ*), sets of clusters with high *λ* were flagged and loci within each SOC were identified as neutral, outlier, or environmentally associated (env) loci. Finally, PCA was run separately for each set of loci from a SOC using *adegenet* to visualize the contribution of those loci to previously observed genetic structuring.

### 2.6 Genetic demographic history

To better understand the demographic history of gafftopsail catfish, a differential approximation of the site frequency spectrum (SFS) was performed using Moments (Jouganous et al., 2017) and the neutral data set. One SNP was randomly selected from each locus and the site frequency spectrum (SFS) down projected using *easySFS* (Gutenkunst et al., 2009) to reduce the effect of missing data and to increase the speed of modeling. Six models were considered using symmetrical migration and population divergence between geographically adjacent populations. Multiple rounds of optimization are required to find the optimal estimates of demographic parameters for each model (Noskova et al., 2020), therefore, the four‐step optimization procedure outlined in Portik et al. (2017) was implemented, with the optimal parameters from the previous step and perturbed at least 40 times. The optimization procedure was then rerun multiple times, using the parameter estimates from the previous run as a starting point, until the AIC values no longer changed. Finally, the model with the lowest AIC value was selected and confidence intervals around point estimates of demographic parameters were generated using the Godambe information matrix (Coffman et al., 2016) implemented in Moments. Final estimates of demographic parameters used a generation time of 10 years and mutation rates of 2.5 × 10^−8^ to produce an upper bound, 3.5 × 10^−9^ to produce a lower bound, and the average (1.43 × 10^−8^) to produce point estimates. Because the genome‐wide mutation rate for ariid catfishes is not known, mutation rates were taken from the literature (Kavembe et al., 2016; Malinsky et al., 2018).

All figures were generated using *ggplot2* (Wickham, 2016) and *UpsetR* (Conway et al., 2017). An Rmarkdown and corresponding rendered html‐document containing reproducible code for the complete analysis and functions as a standalone extended presentation of methods and results can be accessed at https://github.com/marinegenomicslab/gafftop_popgen_2024.

## 3 RESULTS

### 3.1 Genotyping

Initially, 1311, 068 SNPs were identified among 406 individuals. After filtering, the final data set consisted of 367 gafftopsail catfish individuals and 14,682 SNPs. Read depths per SNP were between 15 and 265, with a mean read depth of 78, and read depths per individual were between 10 and 269, with a mean read depth of 78. After haplotyping there were 5554 microhaplotype loci, with 2–25 alleles and 2–18 SNPs per locus. The final data set had less than 15% missing data per population, less than 10% missing data per locus, and less than 23% missing data per individual.

### 3.2 Test for homogeneity and patterns of variance

The single‐level AMOVA for the full data set revealed significant heterogeneity among locations (*F*_ST_ = 0.0123, *p* < .00005, 95% CI 0.0118–0.127). The top three principal components explained 2.53% of the total variance with ATL and CAMP separating from locations in the Gulf on PC1 and PC2 and the Gulf locations separating into the northeastern Gulf (FLGS and FLGN) and northwestern Gulf (MB, MISS, CS, LA and CC) along PC3 (Figure S1).

### 3.3 Identification of neutral and non‐neutral loci

**FIGURE 2:**
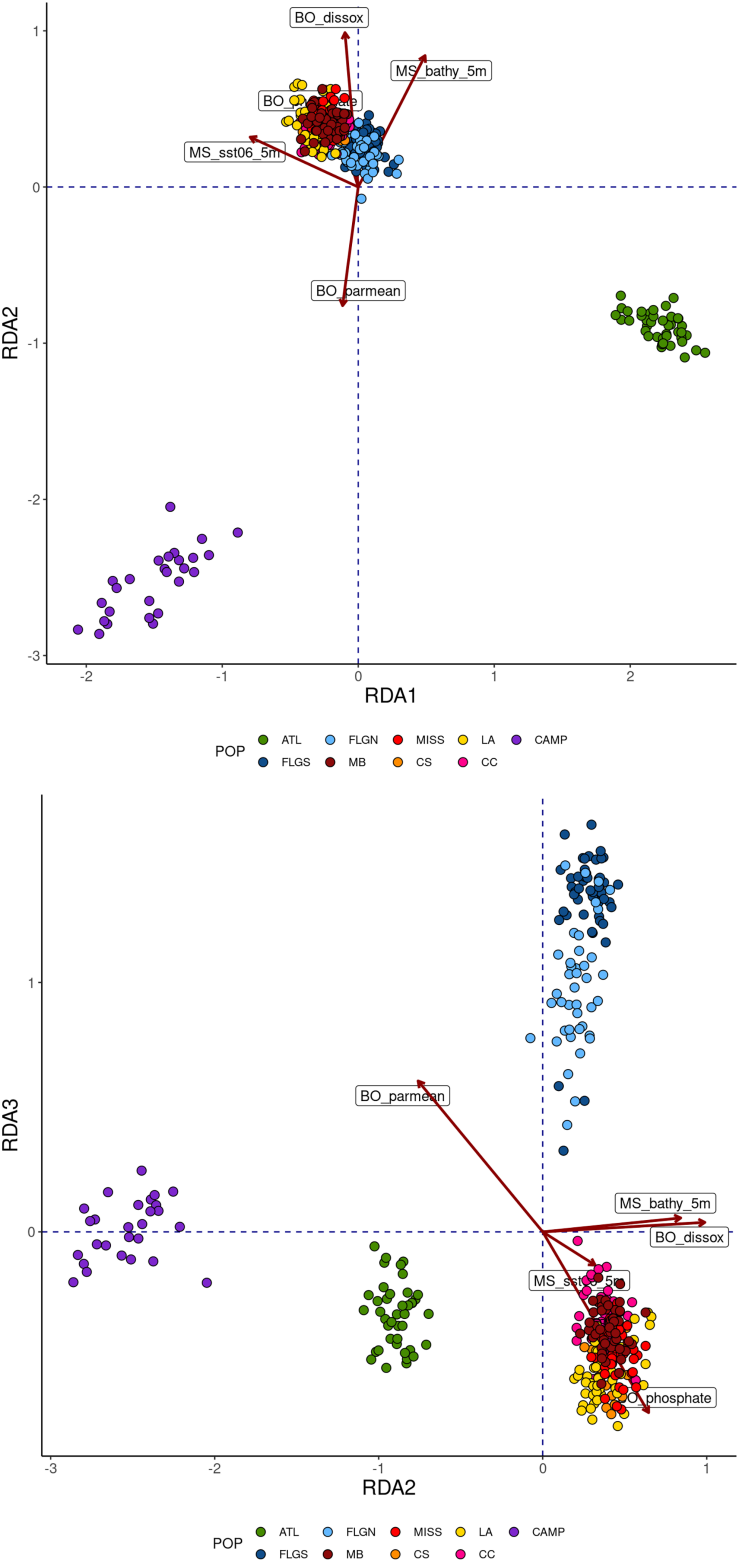
Biplot of redundancy analysis using the environmental model alone. The nine geographic locations are Indian River Lagoon, Florida (ATL) in the Atlantic, Tampa Bay, Florida (FLGS), North of Tampa Bay, Florida (FLGN) Mobile Bay, Alabama (MB), Mississippi Sound, Mississippi (MISS), Chandeleur Sound, LA (CS), off Louisiana west of the Mississippi River (LA), Corpus Christi Bay, Texas (CC), and the Bay of Campeche, Mexico (CAMP). Selected environmental variables are mean sea surface temperature in June (MS_sst06_5m), depth (MS_bathy_5m), dissolved oxygen (BO_dissox), amount of photosynthetically available radiation at the sea surface (BO_parmean), and concentration of ortho‐phosphate (BO_phosphate).

The two outlier detection methods identified 50 outliers (0.9% of all loci) putatively under directional selection. Analysis in arlequin identified 48 outlier loci, while bayescan identified 15, 13 of which were also identified in arlequin. The first four MEMs were selected, as were five environmental variables: mean sea surface temperature in June (MS_sst06_5m), depth (MS_bathy_5m), dissolved oxygen (BO_dissox), amount of photosynthetically available radiation at the sea surface (B0_parmean), and concentration of ortho‐phosphate (BO_phosphate). Both geographic position and environment were significant alone and in combination in the full model, as were shared effects (Table 1), with environment explaining a greater portion of the variance than geographic position. Inspection of the RDA biplot revealed clustering similar to that of the initial PCA, with ATL and CAMP separating from the remaining locations in the Gulf along RDA1 and RDA2 and the northeastern and northwestern Gulf separating along RDA3 (Figure 2). A total of 118 loci had an allele with a Mahalanobis distance ≥20.51 (*p* < .001), with 35 loci matching those previously identified as outliers. For downstream analyses, the full data set was divided into a neutral data set (5421 loci) and a non‐neutral data set (133 loci).

**TABLE 1:** Variance partitioning for geographic position (xy), environmental variables (env), and shared effects (shared) due to interaction of geographic position and environment.

Partition	Variance	*p*‐Value
Residuals	0.9800	NA
xy + env + shared	0.0200	<.001
env + shared	0.0189	<.001
env	0.0121	<.001
xy + shared	0.0078	<.001
shared	0.0068	NA
xy	0.0011	<.001

### 3.4 Genetic variation within and among locations

**FIGURE 3:**
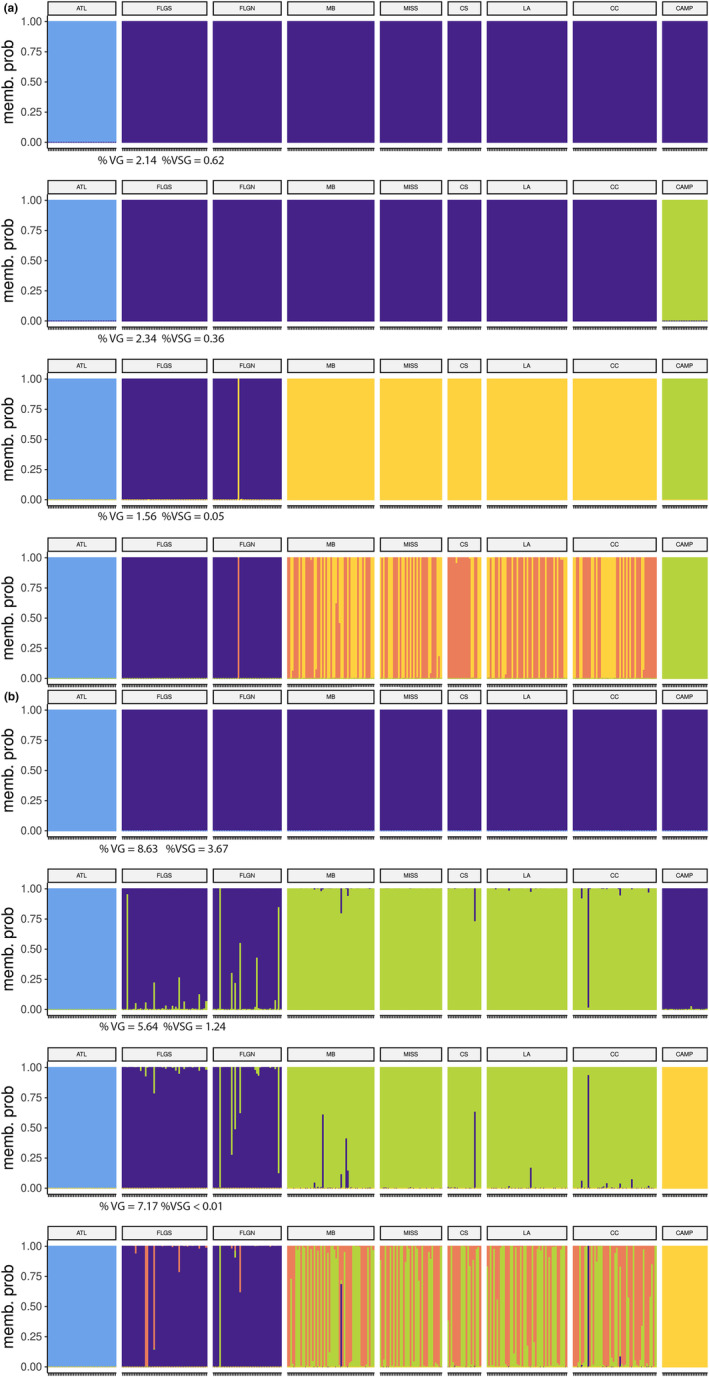
Assignment plots for *K*‐means clustering (*K* = 2–5) for the neutral (a) and outlier (b) data sets. The nine geographic locations are Indian River Lagoon, Florida, in the Atlantic (ATL), Tampa Bay, Florida (FLGS), North of Tampa Bay, Florida (FLGN) Mobile Bay, Alabama (MB), Mississippi Sound, Mississippi (MISS), Chandeleur Sound, LA (CS), off Louisiana west of the Mississippi River (LA), Corpus Christi Bay, Texas (CC), and the Bay of Campeche, Mexico (CAMP). The component of variance (calculated in an AMOVA framework) attributable to differences between groups (%VG) and the component of variance attributable to differences between samples within groups (%VSG) are reported for *K* = 2–4.

For the neutral data set, the minimum AIC value was obtained for *K* = 4 (Figure S2a) and cross‐validation was run for *K* = 2–5. Assignment plots (Figure 3a) revealed hierarchical structure as *K* = 2 divided ATL from all other locations, *K* = 3 split CAMP from the remaining locations; and *K* = 4 further divided the northeastern Gulf (FLGS, FLGN) and northwestern Gulf (MB, MISS, CS, LA, CC). At *K =* 5 assignment success decreased, with no obvious further geographic clustering in the assignment plot. The component of variance attributable to differences between groups was maximized for *K* = 3 (2.34%) but was only slightly larger than for *K* = 2 or 4 (2.14% and 1.56%, respectively), while the component of variance attributable to differences between locations within groups was smaller for *K* = 4 (0.05%) than for *K* = 2 or 3 (0.62% and 0.26%, respectively). Estimates of pairwise *F*_ST_ (Table S1a) were significant after correction for 81% of comparisons (26/36), with small non‐significant estimates between FLGS and FLGN and between 9 out of 10 comparisons in the northwestern Gulf, though MB and LA were significantly different.

For the non‐neutral data set, AIC values continued to decrease with an increase in the number of clusters, but the magnitude of change became relatively small after *K* = 4 (Figure S2b), and cross‐validation was run for *K* = 2–5. Assignment plots (Figure 3b) revealed a hierarchical structure that differed from the neutral data. At *K* = 2, ATL separated from the rest of the locations, while *K* = 3 split FLGS, FLGN, and CAMP from the northwestern Gulf locations, and *K* = 4 split CAMP from the northeastern Gulf. At *K =* 5, assignment success decreased, with no obvious further geographic clustering in the assignment plot. The component of variance attributable to differences between groups was maximized for *K* = 2 (8.63%) and the component of variance attributable to differences between locations within groups was minimized at *K* = 4 (< 0.01%). Estimates of pairwise *F*_ST_ (Table S1b) were significant after correction for 81% of comparisons (26/36), with non‐significant estimates at or near zero between all ten comparisons in the northwestern Gulf; however, FLGN and FLGS were significantly different. Divergence estimates that were significant for both data sets were approximately three to ten times larger in the non‐neutral data set relative to the neutral data set (Table S1c).

Expected heterozygosity differed significantly among the nine locations for both data sets (*p* < .0001). For the neutral data set, estimated *H*_e_ was lowest in ATL (0.269) and greatest in CAMP (0.284; Table 2). After correction, nine pairwise comparisons were significant, with eight comparisons involving ATL (Table 2, Table S2). For the non‐neutral data set, estimated *H*_e_ was lowest in MISS (0.198) and greatest in ATL (0.349, Table 2). After correction, ten comparisons were significant, with seven involving ATL and four involving MISS (Table 2, Table S3). Allelic richness also differed significantly among the nine locations for both data sets (*p* < .0001). For the neutral data set, the estimated *A*_r_ was lowest in ATL (2.274) and highest in CAMP (2.586), and estimates for northwestern Gulf locations were uniformly higher than for northeastern Gulf locations (Table 2). After correction, 30 pairwise comparisons were significant (Table S3), with ATL less diverse and CAMP more diverse than all other locations. For the non‐neutral data set, the estimated *A*_r_ was lowest in MISS (1.94) and greatest in ATL (2.161, Table 2). After correction, five pairwise comparisons were significant (Table S3), with ATL and CS more diverse than LA and MISS, and CAMP more diverse than LA.

**TABLE 2:** Estimates of expected heterozygosity (*H*_e_) and rarified allelic richness (*A*_r_) for the nine geographic samples (locations) using neutral (Neu) and non‐neutral (NN) data sets. The nine geographic locations are Indian River Lagoon, Florida (ATL) in the Atlantic, Tampa Bay, Florida (FLGS), North of Tampa Bay, Florida (FLGN) Mobile Bay, Alabama, (MB), Mississippi Sound, Mississippi (MISS), Chandeleur Sound, LA (CS), off Louisiana west of the Mississippi River (LA), Corpus Christi Bay, Texas (CC), and the Bay of Campeche, Mexico (CAMP). The number of significant pairwise comparisons in which a location was higher (+) or lower (−) are indicated.

*H*_e_	Neu	+	−	NN	+	−
ATL	0.269	0	8	0.349	7	0
FLGS	0.281	1	1	0.252	1	1
FLGN	0.282	1	0	0.255	1	1
MB	0.282	2	0	0.213	0	1
MISS	0.282	1	0	0.198	0	4
CS	0.283	1	0	0.218	0	1
LA	0.282	1	0	0.211	0	1
CC	0.282	1	0	0.218	0	1
CAMP	0.284	1	0	0.274	1	0

*A*_r_	Neu	+	−	NN	+	−
ATL	2.274	0	8	2.161	2	0
FLGS	2.461	1	7	2.074	0	0
FLGN	2.468	2	6	2.123	0	0
MB	2.492	4	2	2.003	0	0
MISS	2.489	4	1	1.938	0	2
CS	2.469	3	4	2.053	2	0
LA	2.487	4	1	1.965	0	3
CC	2.496	4	1	1.992	0	0
CAMP	2.586	8	0	2.139	1	0

**FIGURE 4:**
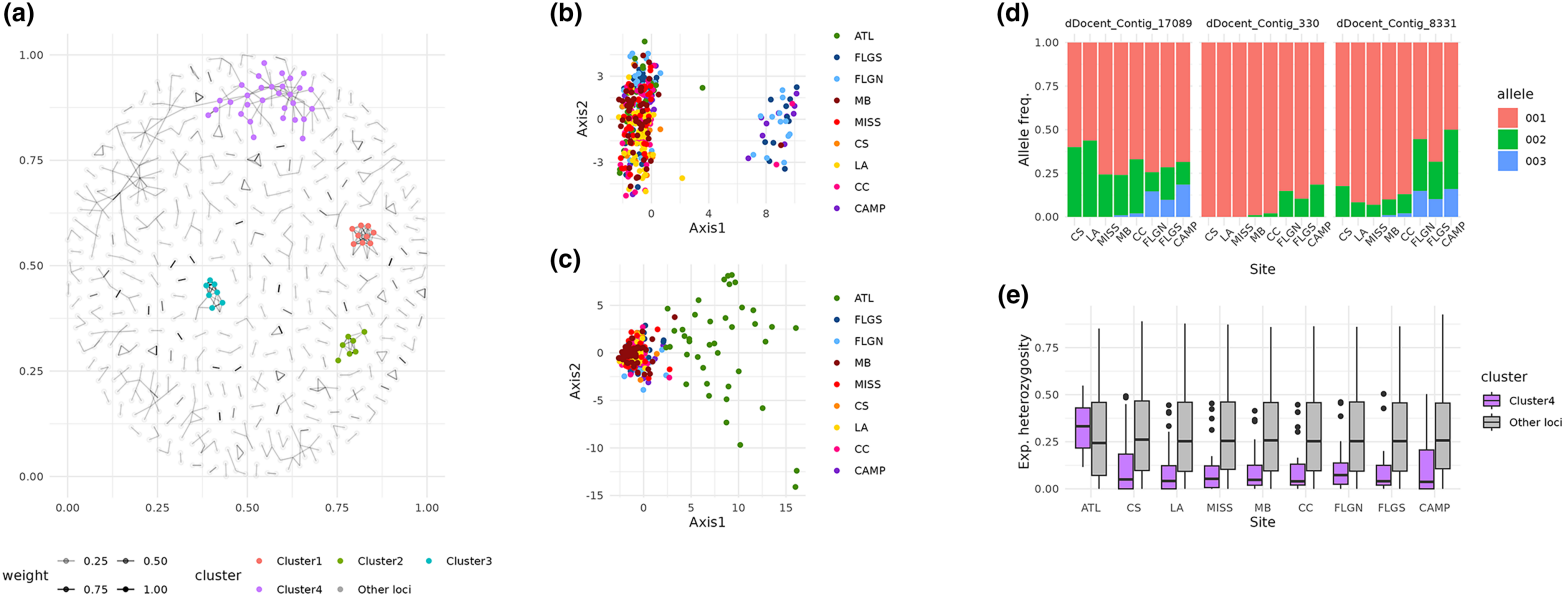
Results of linkage disequilibrium network analysis showing the four distinct clusters (a), with PCAs for loci in Cluster 3 (b) and Cluster 4 (c), as well as representative allele frequency plots for 3 of the 5 outlier/env loci in Custer 3 (d) and a comparison of expected heterozygosity for 15 outlier/env loci in Cluster 4 with expected heterozygosity at all other loci for each location (e). The nine geographic locations are Indian River Lagoon, Florida, in the Atlantic (ATL), Tampa Bay, Florida (FLGS), North of Tampa Bay, Florida (FLGN) Mobile Bay, Alabama (MB), Mississippi Sound, Mississippi (MISS), Chandeleur Sound, LA (CS), off Louisiana west of the Mississippi River (LA), Corpus Christi Bay, Texas (CC), and the Bay of Campeche, Mexico (CAMP).

For LDna analysis, the plot of *λ* values appeared to be asymptotic; therefore, a threshold value near the inflection point was selected (Figure S3). Four SOCs were identified (Figure 4a; Figure S3), two of which contained only neutral loci, with nine loci in Cluster 1 and eight loci in Cluster 2. The other two clusters contained a mix of neutral, outlier, and env loci. Cluster 3 contained two neutral loci, four outlier, and five env loci (four loci overlapped between outlier and env loci). Cluster 4 contained 15 neutral loci, 14 outlier, and 11 env loci (ten loci overlapped between outlier and env loci). The PCA plots using loci from Cluster 3 and Cluster 4 were in stark contrast, with the plot resulting from Cluster 3 showing the northeastern Gulf and CAMP pulling away from the other regions (Figure 4b), while the plot resulting from Cluster 4 showed ATL separating from all other regions (Figure 4c). Allele frequencies at non‐neutral loci from Cluster 3 displayed a latitudinal pattern in the Gulf, with more alleles present at lower latitudes (Figure 4d). By contrast, non‐neutral loci from Cluster 4 had relatively similar numbers of alleles across geographic samples but displayed elevated *H*_e_ in ATL relative to all other locations (Figure 4e).

### 3.5 Genetic demographic history

**FIGURE 5:**
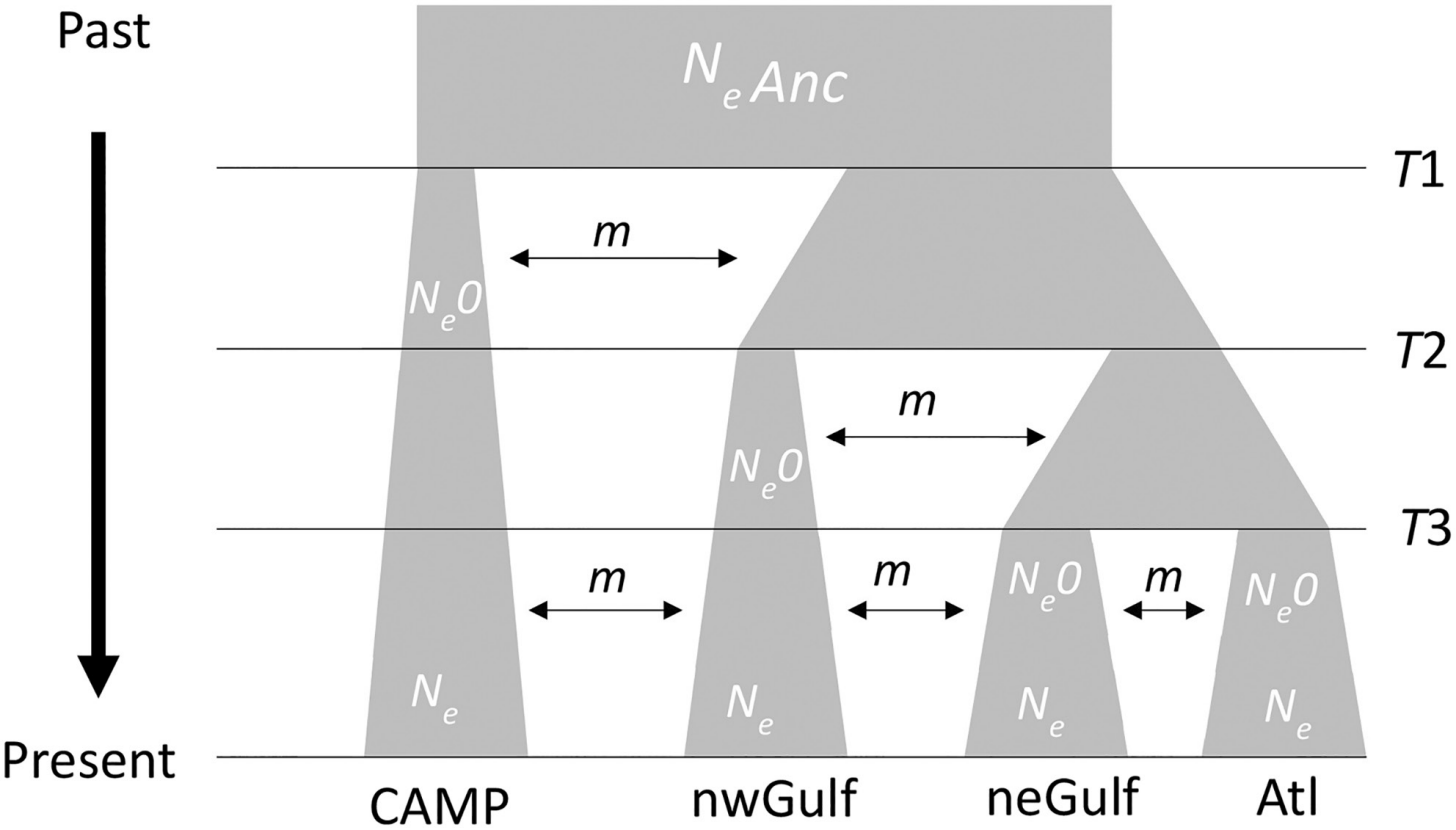
Model 3 selected using site frequency spectrum analysis. Parameters of the model include ancestral long‐term effective size (*N*_e_Anc), long‐term effective size post‐split (*N*_e_0), current long‐term effective size (*N*_e_), split times (*T*), and symmetrical migration rates (*m*). The four populations are Campeche in the southern Gulf of Mexico (CAMP), the northwestern Gulf of Mexico (nwGulf), the northeastern Gulf of Mexico (neGulf), and the U.S. Atlantic (Atl).

Model three (Figure 5), which had the lowest AIC (Table 3), identified CAMP as separating from all other populations first (*T*1), followed by the northwestern Gulf (*T*2), with a more recent split (*T*3) between the northeastern Gulf and the Atlantic. All other models are shown in Figure S4. Estimates of split times for *T*1 (159,226 ybp; 87,314–677,432) and *T*2 (158,586 ybp; 87,005–674,415) were similar, while the estimate of T3 (17 ybp; 10–73) was much more recent. Estimated current long‐term *N*_e_ was largest for the northwestern Gulf (129,683; 62,701–611,829), followed by the CAMP (7369; 15,558–74,494) and the northeastern Gulf (926; 500–3993), with a relatively small estimate for the Atlantic (10; 6–43). Point estimates of current long‐term *N*_e_ for the Atlantic and northeastern Gulf were also relatively smaller than estimates of long‐term *N*_e_ post‐split (Atlantic, *N*_e_ = 22; northeastern Gulf; *N*_e_ = 13,960). Parameter estimates are shown in Table S4.

**TABLE 3:** Results of site frequency spectrum model analysis showing the number of optimization rounds required (Opt), the log likelihood values (LnL), as well as the Akaike Information criteria values (AIC), and their change (ΔAIC).

Model	Opt	LnL	AIC	ΔAIC
model_3	9	−2787.2	5614.3	0
model_2	11	−6376.8	12,795.7	−7181
model_1	10	−6416.9	12,875.8	−7261
model_4	5	−6631.7	13,299.4	−7685
model_5	6	−6822.1	13,684.2	−8070
Null	2	−8455.3	16,920.6	−11,306

## 4 DISCUSSION

Patterns of genetic variation among gafftopsail catfish sampled at eight locations across the Gulf of Mexico and one in the U.S. Atlantic were assessed using over 5000 microhaplotypes. Analyses using datasets of neutral and non‐neutral loci recovered four independent genetic units that corresponded to the samples collected in the Atlantic (ATL), the northeastern Gulf of Mexico (FLN + FLS), the northwestern Gulf (MB, MISS, CS, LA, and CC) and the Bay of Campeche (CAMP); however, hierarchical patterns of structuring differed among the data sets. Expected heterozygosity was lower for ATL than all other locations for neutral loci, but higher for non‐neutral loci. Allelic richness was lowest in ATL and highest in CAMP for neutral loci and generally showed higher values moving from west to east and north to south in the Gulf for non‐neutral loci. Linkage disequilibrium network analysis detected two clusters involving multiple outlier/environmental loci. The first cluster had five loci, with allelic gradients changing from west to east and north to south, consistent with patterns of within‐group diversity. By contrast, the second cluster featured loci that showed elevated heterozygosity, but not allelic richness, in ATL relative to all other locations. Furthermore, demographic analyses suggest that patterns of variation in the northeastern Gulf and ATL may have been impacted by relatively recent declines in population size.

The neutral population structure exhibited in this study is congruent with what has been observed in a variety of nearshore/estuarine fishes along the U.S. Atlantic and Gulf Coast, in terms of the regional groupings recovered and the inferred location of geographic boundaries for those groups (sheepshead, *Archosargus probatocephalus*, Seyoum et al., 2017; spotted seatrout, *Cynoscion nebulosus*, Seyoum et al., 2018; red drum, *Sciaenops ocellatus*, Hollenbeck et al., 2019). The clear divergence of Atlantic gafftopsail catfish from all Gulf samples (Figures 2 and 3) is consistent with distributional data; gafftopsail catfish are found in high numbers along the Gulf Coast of Florida and historically on the Atlantic Coast of Florida from Indian River Lagoon north, but more rarely encountered in coastal southeastern Florida and the Keys (Armstrong et al., 1996; Serafy et al., 2003). This break in distribution coincides with the Florida Vicariance Zone (Neigel, 2009), an area defined by a narrowing of the continental shelf and subsequent reduction/absence of estuarine habitat south of West Palm Beach, Florida, which is influenced by the swift‐moving surface currents in the Florida Straits (Lynch‐Stieglitz & Slowey, 1999). While it has been suggested that the east‐flowing Florida Current may facilitate larval connectivity from west to east for discontinuously distributed species (e.g., Karnauskas et al., 2022), for species like gafftopsail catfish that lack a dispersive larval phase the area may represent a hard barrier (Portnoy et al., 2014). Consistent with this idea, pairwise differences between the ATL and northeastern Gulf locations were of similar magnitude to differences between the ATL and northwestern Gulf locations and largely decoupled from distance (Table S1).

Divergence between CAMP and all other locations also is consistent with distributional data. The species is commonly encountered in the Bay of Campeche and along the Texas Coast, but less frequently encountered on the Yucatan platform and along portions of the Tamaulipas and Veracruz coastlines in Mexico, where the shelf narrows and estuarine habitat is reduced (DOF, 2018). In addition, while the Yucatan Channel and open waters of the Gulf can be traversed by marine fishes during the larval stage, resulting in connectivity between Mexican waters and the West Florida Shelf (Johnston & Bernard, 2017), this area likely proves a more effective barrier for gafftopsail catfish.

The divide between the northeastern and the northwestern Gulf falls within a well characterized biogeographic break centered on Mobile Bay, Alabama (McClure & McEachran, 1992), which has been attributed to a variety of current and historical processes (Portnoy & Gold, 2012) and is consistent with genetic breaks observed in a number of estuarine dependent species, including live bearing sharks and broadcast spawning bony fish with a larval phase (e.g., Hollenbeck et al., 2019; Portnoy et al., 2016; Seyoum et al., 2018). For gafftopsail catfish, estimated levels of divergence between northeastern and northwestern Gulf samples were approximately five times smaller than what was seen between northern Gulf samples and CAMP or ATL suggesting that the divergence is more recent and/or levels of recurrent gene flow persist across the northern Gulf, as the species appears continuously distributed in the region. Though there was no strong evidence of admixture in the data, the latter possibility is consistent with the idea that the central northern Gulf represents a marine suture zone, as has been suggested for red drum (Hollenbeck et al., 2019).

Site frequency spectra analysis provided insight into how historical demography has contributed to patterns of contemporary variation. Estimated split times for CAMP and the northwestern Gulf were very similar, falling largely within the Pleistocene (2.6 my – 11.8 kya), during which there were repeated glaciation events (see Ehlers & Gibbard, 2008 for a review), with point estimates in the glacial period that ended ~130 kya (Hughes & Gibbard, 2018). A combination of changes in salinity, reduced sea surface temperature, and decreased availability of nearshore habitat due to sea level decline (Bruner, 1982; Simms et al., 2007) may periodically have made portions of the northern Gulf inhospitable for estuarine‐dependent species like gafftopsail catfish. This may have caused periodic retreats into glacial refugia (Provan & Bennett, 2008) that lead to population subdivision via drift and selection, a scenario that has already been hypothesized to be an important driver of contemporary patterns of variation across taxa in the Gulf (Hollenbeck et al., 2019; Portnoy et al., 2014). By contrast, the estimated split time between ATL and the northeastern Gulf was relatively recent (<73 ya) and the estimate of long‐term effective size of ATL very small (<43), suggesting that accelerated drift processes in ATL may have a large impact on current patterns of variation. Furthermore, the results indicate declines in long term effective size for both the northeastern Gulf (~15X based on point estimates) and ATL (~2X based on point estimates). The latter result is consistent with the documented declines in abundance of gafftopsail catfish along the U.S. Atlantic coast beginning in the late 1990's that are potentially attributable to “hardhead catfish virus” which caused documented massive mortality events for the hardhead catfish, *Ariopsis felis*, in the Atlantic around the same time period (Overstreet & Hawkins, 2017; Webster, 2015).

A comparison of the neutral and non‐neutral datasets suggests that localized adaptation driven by regional environmental differences may play an important role in maintaining patterns of neutral structure (Nosil et al., 2008). While both data sets recovered the same four genetic units (ATL, northeastern Gulf, northwestern Gulf, and CAMP), patterns of hierarchal structuring, explored through *K*‐means clustering, were not congruent. In addition, estimates of *F*_ST_ were larger for the non‐neutral dataset, which is to be expected, but post hoc Mantel testing indicated that patterns between the datasets were not significantly correlated (*p* > .05), with the magnitude of increase greatest for comparisons between northeastern Gulf and northwestern Gulf samples (Table S1c). This reinforces the notion that the non‐neutral loci are not simply drift outliers. This idea is further supported by estimates of diversity within groups. Estimates of *H*_e_ and *A*_r_ were high in FLGS, FLGN, and CAMP relative to northwestern Gulf samples for non‐neutral loci, suggesting strong directional selection in the western Gulf or diversifying selection in the eastern Gulf. Given that the western Gulf population extends to Mobile Bay, well past the Mississippi River, and the lack of another obvious physical barrier, the results suggest isolation by adaptation dynamic (Orsini et al., 2013), which has been suggested for a variety of species where divergence is associated with differences in habitat that occur on scales well within species dispersal ranges (Bond et al., 2014; Hollenbeck et al., 2022; Jiang et al., 2019). Similar patterns of divergence and spatial structuring in neutral and non‐neutral loci are seen in the co‐distributed red drum (Hollenbeck et al., 2019), suggesting that selection may be operating on both species in a similar way. In this study, two sets of non‐neutral loci were observed, those that distinguished the southern and northeastern Gulf from the northwestern Gulf, corresponding to shifts in allele frequencies, and those that separated the Atlantic from all other samples, characterized by elevated expected heterozygosity in the Atlantic (Figure 4c,e).

Directional selection associated with environmental heterogeneity can be an important driver of contemporary patterns of genetic structure (Nosil et al., 2009). In this study, five environmental factors were identified as significantly correlated with components of genetic variation and three (mean sea surface temperature in June, amount of photosynthetically available radiation at the sea surface, and concentration of ortho‐phosphate) are associated with environmental parameters, like temperature and rainfall, that vary with latitude in the northeastern Gulf of Mexico (Hollenbeck et al., 2019). For species in the northern hemisphere, lower latitudes were less impacted by recent glacial cycles and thus represent relatively stable environments over evolutionary time scales; therefore, populations at low latitudes may display greater overall levels of genetic variation at genome‐wide scales (Adams & Hadly, 2013; Hasselman et al., 2013). In the case of gafftopsail catfish, however, increased diversity with latitude is present at a subset of loci (though the southern Gulf features relatively high neutral diversity as well) consistent with latitude‐associated selection, something that has been detected in other marine taxa (e.g., fishes: Bradbury et al., 2010; corals: Thomas et al., 2017; bivalves: Vendrami et al., 2019), including in the Gulf of Mexico (sharks: Portnoy et al., 2015).

Heterozygote advantage, which can result from numerous causes including increased enzymatic capacity and increased immune function, has not been frequently documented in wild systems (Hedrick, 2012), but in such cases is almost always associated with immune function (Gemmell & Slate, 2006). Increased immune function is an intriguing possibility for gafftopsail catfish in the Atlantic because the species, once very common, experienced a rapid decline in abundance (>90%) beginning in the mid‐1990s, a phenomenon potentially caused by a pathogen (Webster, 2015). In this situation, perhaps strong selection for immune diversity, associated with increased functionality for pathogen resistance (Evans & Neff, 2009; Osborne et al., 2015) may have created the pattern observed in the data. There are no appropriate genomic resources currently available for arid catfishes that can be used to support or refute either idea. Furthermore, because of the species continued rarity along the Atlantic Coast of the U.S., this study was only able to acquire a sufficient number of individuals from one U.S. Atlantic location in Florida. Thus, it is not known whether the patterns seen in the genomic data are coast‐wide or locality‐specific and further research would be needed to explore this possibility.

## 5 CONCLUSIONS

A common theme in the marine population genetics literature is understanding the effect that larval dispersal has on patterns of spatial genetic structure (Faurby & Barber, 2012). Because gafftopsail catfish are a directly developing species in which males brood the young and deliver them into estuarine habitat, they are an interesting comparison to most bony fishes with dispersive larval phases. Patterns of structure found here in gafftopsail catfish were very similar to what is seen for several bony fishes in the Gulf of Mexico and U.S. South Atlantic that have larval phases but, like gafftopsail catfish, are estuarine obligates for part of their life cycle (see Hollenbeck et al., 2019). Furthermore, similar patterns of structure have been documented in several shark species that use estuaries as nursery habitat (Portnoy et al., 2014, 2016; Swift et al., 2023). By contrast, bony fish species that use less consolidated shelf habitat (e.g., red snapper, *Lutjanus campechanus*) show less structure over the same geographic area (see Portnoy et al., 2022). Taken together, this adds to the argument that aspects of adult/juvenile behavior (including dispersal) as well as patterns of habitat use are likely important but neglected predictors of the scale and pattern of population structure in marine species (Portnoy et al., 2022).

While elucidating the underlying mechanisms will require further work, this study demonstrated that patterns of diversity at non‐neutral loci were attributable to different evolutionary processes. Structure associated with non‐neutral loci should be complex in wild systems because interactions between genes and the environment differ in time, space, and across genomes (White & Butlin, 2021), yet many studies treat selection as unidimensional. Access to genomic resources, such as whole genomes or linkage maps, will allow researchers to characterize the distribution of differences across genomes within and across populations (the genomic landscape of divergence, Quilodrán et al., 2020), which is important for understanding more complicated evolutionary scenarios and investigating forces such as recombination that have been historically underexplored. The development of such resources is a clear next step toward understanding the adaptive variation presented in this study.

## AUTHOR CONTRIBUTIONS

**David S. Portnoy:** Conceptualization (lead); formal analysis (equal); funding acquisition (equal); project administration (lead); visualization (equal); writing – original draft (lead); writing – review and editing (equal). **Shannon J. O'Leary:** Conceptualization (equal); data curation (equal); formal analysis (equal); visualization (equal); writing – original draft (supporting); writing – review and editing (equal). **Andrew T. Fields:** Data curation (equal); formal analysis (equal); visualization (equal); writing – original draft (supporting); writing – review and editing (equal). **Christopher M. Hollenbeck:** Data curation (equal); formal analysis (equal); visualization (equal); writing – original draft (equal); writing – review and editing (equal). **R. Dean Grubbs:** Conceptualization (supporting); resources (equal); writing – review and editing (equal). **Cheston T. Peterson:** Resources (supporting); writing – review and editing (supporting). **Jayne M. Gardiner:** Resources (equal); writing – review and editing (supporting). **Douglas H. Adams:** Resources (equal); writing – review and editing (equal). **Brett Falterman:** Resources (equal); writing – review and editing (supporting). **J. Marcus Drymon:** Resources (equal); writing – review and editing (supporting). **Jeremy M. Higgs:** Resources (equal); writing – review and editing (supporting). **Erin L. Pulster:** Resources (supporting); writing – review and editing (supporting). **Tonya R. Wiley:** Resources (equal); writing – review and editing (supporting). **Steven A. Murawski:** Conceptualization (equal); funding acquisition (lead); resources (equal); writing – review and editing (equal).

## CONFLICT OF INTEREST STATEMENT

The authors have no conflict of interest to declare.

## Supporting information


Appendix S1.

## Data Availability

Individual SNP genotypes, custom scripts, and filtering details are available at https://github.com/marinegenomicslab/gafftop_popgen_2024. Metadata is publicly available through the Gulf of Mexico Research Initiative Information & Data Cooperative (GRIIDC) at https://data.gulfresearchinitiative.org (doi: [10.7266/n7‐8jya‐4m57]). Raw sequence reads under in the NCBI Sequence Read Archive (accession numbers: SRR25523260‐SRR25523626). The final microhaplotype dataset, in VCF format, and the neutral and outlier haplotype datasets, in genepop format, are available at Dryad doi: 10.5061/dryad.nvx0k6f0n. Benefits from this research revolve around sharing the results in presentations and publications and the data on public databases as described above. The work also involved postdocs, graduate, and undergraduate students who received professional training through their efforts.
