# Supplementary material for: Complex patterns of genetic population structure in the mouthbrooding marine catfish, Bagre marinus, in the Gulf of Mexico and U.S. Atlantic
Source: Ecol Evol. 2024 Jun 9;14(6):e11514. doi: 10.1002/ece3.11514 (PMC11163162; doi:10.1002/ece3.11514)
Supplement: Supplementary file 1 — Appendix S1. [file ECE3-14-e11514-s001.docx]

**Appendix**

Supplemental Figure 1: Results of Principle Components analysis on the full data set. The nine geographic locations are Indian River Lagoon, Florida in the Atlantic (ATL), Tampa Bay, Florida (FLGS), North of Tampa Bay, Florida (FLGN) Mobile Bay, Alabama, (MB), Mississippi Sound, Mississippi (MISS), Chandeleur Sound, LA (CS), off Louisiana west of the Mississippi River (LA), Corpus Christi Bay, Texas (CC), and the Bay of Campeche, Mexico (CAMP).


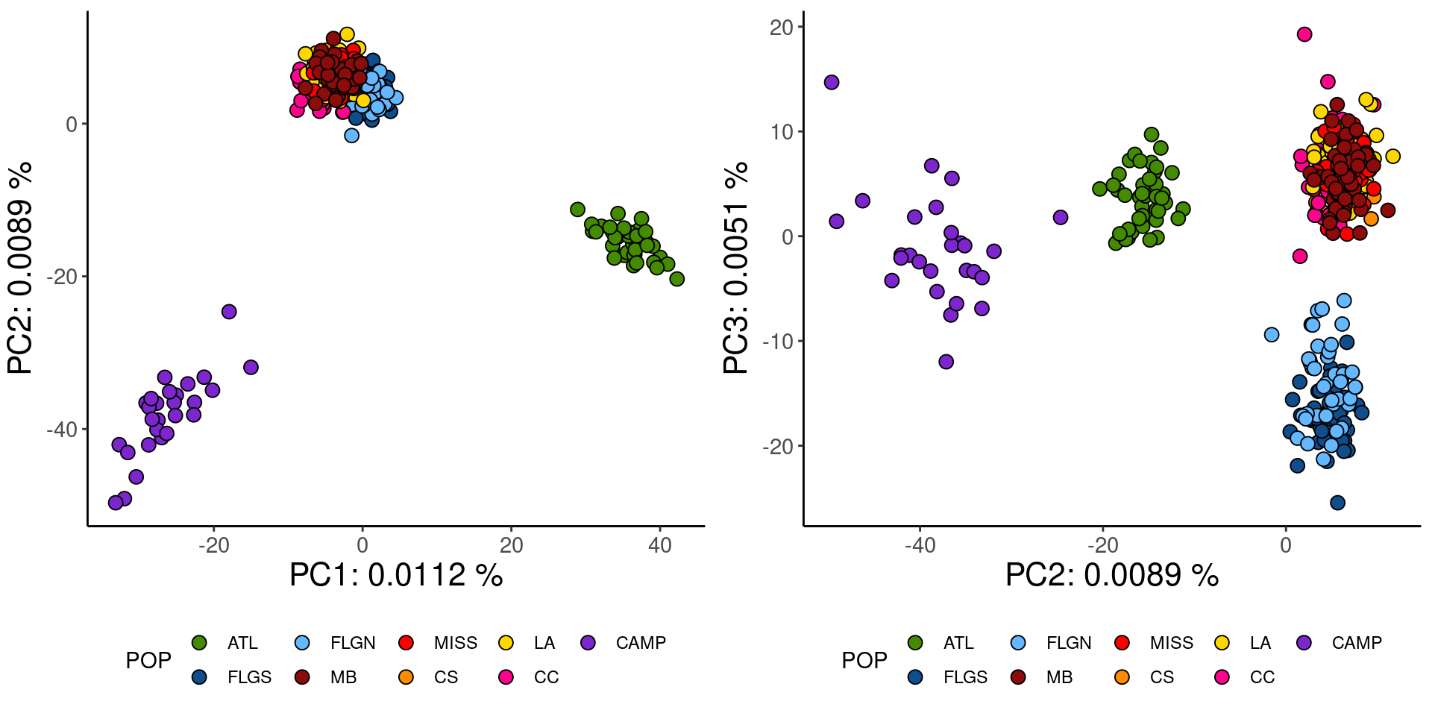


Supplemental Figure 2: Results of *K-*means clustering for the neutral (a), and non-neutral (b) data sets. Results for *K* = 1-10 are presented with Akaike information criterion **(**AIC) values on the y axis.

a.

b


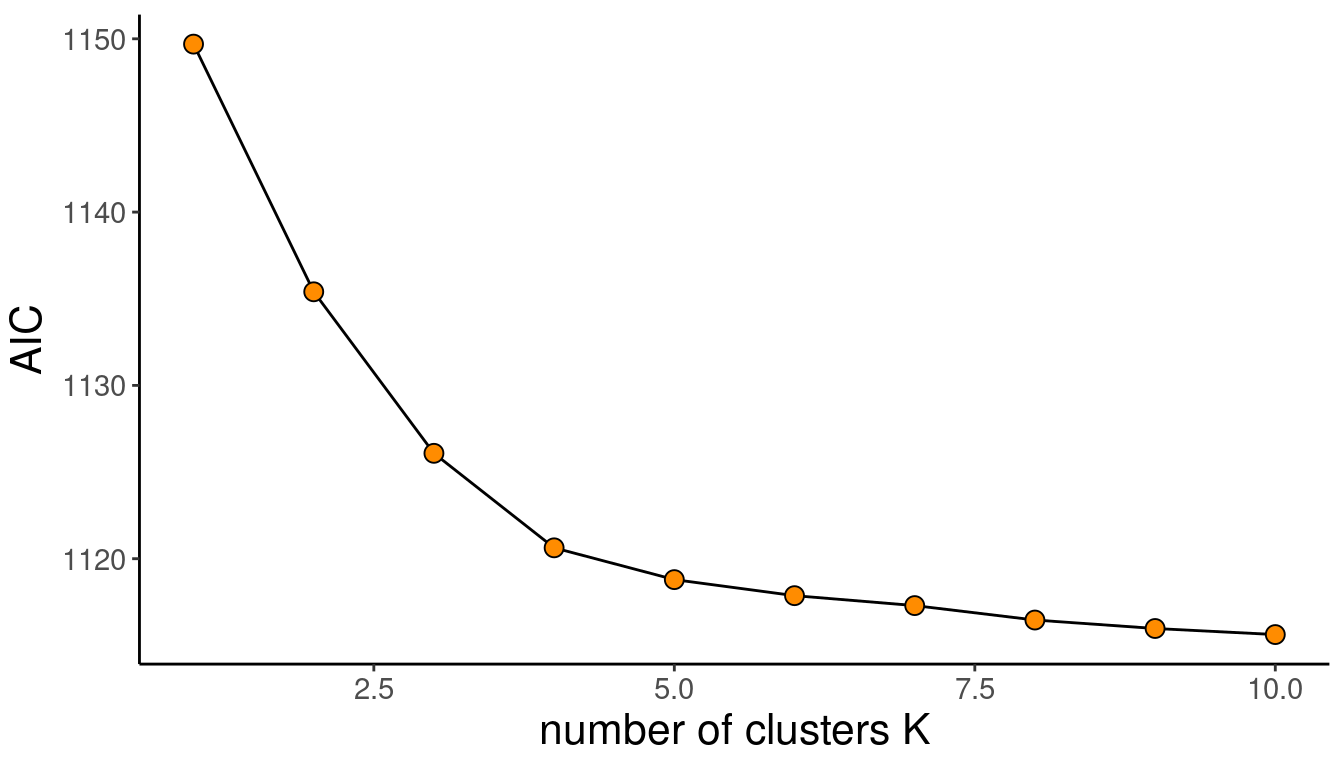


Supplemental Figure 3: Plot of λ values obtained from linkage disequilibrium network analysis. Red dashed lines indicate the threshold value of λ used to define sets of clusters. Lambda values are plotted by rank (index).


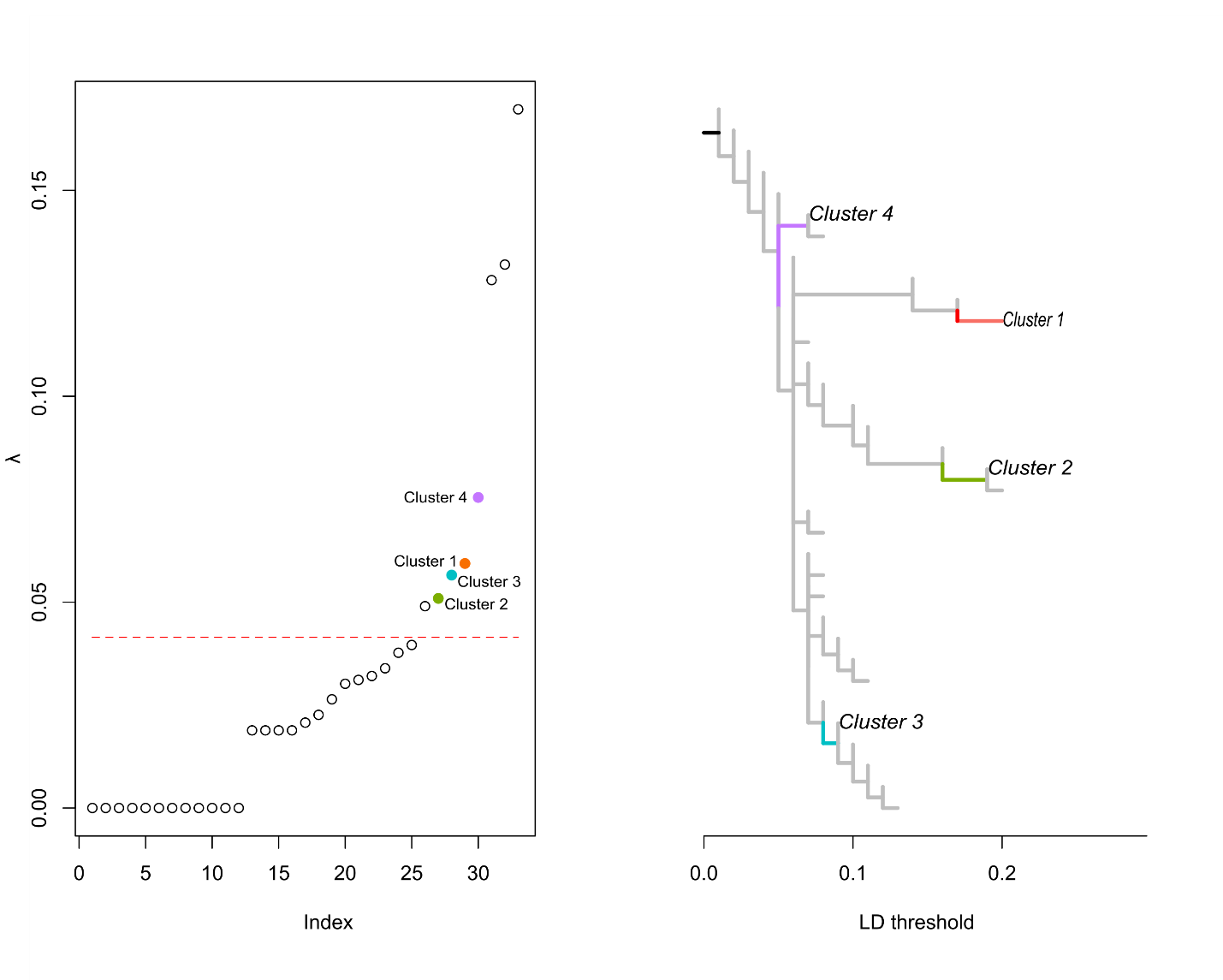


Supplemental Figure 4: Models not selected using site frequency spectrum analysis. The four populations are Campeche in the southern Gulf of Mexico (CAMP), the northwestern Gulf of Mexico (nwGulf), the northeastern Gulf of Mexico (neGulf), and the U.S. Atlantic (Atl)

Present

Past

Null Model

CAMP

nwGulf

neGulf

Atl

CAMP

nwGulf

neGulf

Atl

Present

Past

Model 1

CAMP

nwGulf

neGulf

Atl

Present

Past

Model 2

CAMP

nwGulf

neGulf

Atl

Present

Past

Model 4

CAMP

nwGulf

neGulf

Atl

Present

Past

Model 5

Supplemental Table 1: List of 68 environmental variables initially included in redundancy analysis (RDA) downloaded from Bio-ORACLE or MARSPEC.

| **dataset_code** | **layer_code** | **name** | **units** |
| --- | --- | --- | --- |
| Bio-ORACLE | BO_calcite | Calcite (mean) | mol/m^3 |
| Bio-ORACLE | BO_chlomax | Chlorophyll A (maximum) | mg/m^3 |
| Bio-ORACLE | BO_chlomean | Chlorophyll A (mean) | mg/m^3 |
| Bio-ORACLE | BO_chlomin | Chlorophyll A (minimum) | mg/m^3 |
| Bio-ORACLE | BO_chlorange | Chlorophyll A (range) | mg/m^3 |
| Bio-ORACLE | BO_cloudmax | Cloud fraction (maximum) | % |
| Bio-ORACLE | BO_cloudmean | Cloud fraction (mean) | % |
| Bio-ORACLE | BO_cloudmin | Cloud fraction (minimum) | % |
| Bio-ORACLE | BO_damax | Diffuse attenuation coefficient at 490 nm (maximum) | m^-1 |
| Bio-ORACLE | BO_damean | Diffuse attenuation coefficient at 490 nm (mean) | m^-1 |
| Bio-ORACLE | BO_damin | Diffuse attenuation coefficient at 490 nm (minimum) | m^-1 |
| Bio-ORACLE | BO_dissox | Dissolved oxygen | ml/l |
| Bio-ORACLE | BO_nitrate | Nitrate | micromol/L |
| Bio-ORACLE | BO_parmax | Photosynthetically available radiation (maximum) | Einstein/m_/day |
| Bio-ORACLE | BO_parmean | Photosynthetically available radiation (mean) | Einstein/m_/day |
| Bio-ORACLE | BO_ph | pH | unitless |
| Bio-ORACLE | BO_phosphate | Phosphate | micromol/L |
| Bio-ORACLE | BO_salinity | Salinity | PSS |
| Bio-ORACLE | BO_silicate | Silicate | micromol/L |
| Bio-ORACLE | BO_sstmax | Sea surface temperature (maximum) | Celsius |
| Bio-ORACLE | BO_sstmean | Sea surface temperature (mean) | Celsius |
| Bio-ORACLE | BO_sstmin | Sea surface temperature (minimum) | Celsius |
| Bio-ORACLE | BO_sstrange | Sea surface temperature (range) | Celsius |
| Bio-ORACLE | BO_bathymin | Bathymetry (minimum) | meters |
| Bio-ORACLE | BO_bathymax | Bathymetry (maximum) | meters |
| Bio-ORACLE | BO_bathymean | Bathymetry (mean) | meters |
| MARSPEC | MS_bathy_5m | Bathymetry | meters |
| MARSPEC | MS_biogeo01_aspect_EW_5m | East/West aspect | radians |
| MARSPEC | MS_biogeo02_aspect_NS_5m | North/South Aspect | radians |
| MARSPEC | MS_biogeo03_plan_curvature_5m | Plan curvature | unitless |
| MARSPEC | MS_biogeo04_profile_curvature_5m | Profile curvature | unitless |
| MARSPEC | MS_biogeo05_dist_shore_5m | Distance to shore | kilometers |
| MARSPEC | MS_biogeo06_bathy_slope_5m | Bathymetric slope | degrees |
| MARSPEC | MS_biogeo07_concavity_5m | Concavity | degrees |
| MARSPEC | MS_biogeo08_sss_mean_5m | Sea surface salinity (annual mean) | psu |
| MARSPEC | MS_biogeo09_sss_min_5m | Sea surface salinity (monthly minimum) | psu |
| MARSPEC | MS_biogeo10_sss_max_5m | Sea surface salinity (monthly maximum) | psu |
| MARSPEC | MS_biogeo11_sss_range_5m | Sea surface salinity (annual range) | psu |
| MARSPEC | MS_biogeo12_sss_variance_5m | Sea surface salinity (annual variance) | psu |
| MARSPEC | MS_biogeo13_sst_mean_5m | Sea surface temperature (annual mean) | Celsius |
| MARSPEC | MS_biogeo14_sst_min_5m | Sea surface temperature (coldest ice-free month) | Celsius |
| MARSPEC | MS_biogeo15_sst_max_5m | Sea surface temperature (warmest ice-free month) | Celsius |
| MARSPEC | MS_biogeo16_sst_range_5m | Sea surface temperature (range) | Celsius |
| MARSPEC | MS_biogeo17_sst_variance_5m | Sea surface temperature (variance) | Celsius |
| MARSPEC | MS_sss01_5m | Sea surface salinity (january) | psu |
| MARSPEC | MS_sss02_5m | Sea surface salinity (february) | psu |
| MARSPEC | MS_sss03_5m | Sea surface salinity (march) | psu |
| MARSPEC | MS_sss04_5m | Sea surface salinity (april) | psu |
| MARSPEC | MS_sss05_5m | Sea surface salinity (may) | psu |
| MARSPEC | MS_sss06_5m | Sea surface salinity (june) | psu |
| MARSPEC | MS_sss07_5m | Sea surface salinity (july) | psu |
| MARSPEC | MS_sss08_5m | Sea surface salinity (august) | psu |
| MARSPEC | MS_sss09_5m | Sea surface salinity (september) | psu |
| MARSPEC | MS_sss10_5m | Sea surface salinity (october) | psu |
| MARSPEC | MS_sss11_5m | Sea surface salinity (november) | psu |
| MARSPEC | MS_sss12_5m | Sea surface salinity (december) | psu |
| MARSPEC | MS_sst01_5m | Sea surface temperature (january) | Celsius |
| MARSPEC | MS_sst02_5m | Sea surface temperature (february) | Celsius |
| MARSPEC | MS_sst03_5m | Sea surface temperature (march) | Celsius |
| MARSPEC | MS_sst04_5m | Sea surface temperature (april) | Celsius |
| MARSPEC | MS_sst05_5m | Sea surface temperature (may) | Celsius |
| MARSPEC | MS_sst06_5m | Sea surface temperature (june) | Celsius |
| MARSPEC | MS_sst07_5m | Sea surface temperature (july) | Celsius |
| MARSPEC | MS_sst08_5m | Sea surface temperature (august) | Celsius |
| MARSPEC | MS_sst09_5m | Sea surface temperature (september) | Celsius |
| MARSPEC | MS_sst10_5m | Sea surface temperature (october) | Celsius |
| MARSPEC | MS_sst11_5m | Sea surface temperature (november) | Celsius |
| MARSPEC | MS_sst12_5m | Sea surface temperature (december) | Celsius |

Supplemental Table 2: Estimates of pairwise *F*_ST_ (below the diagonal) and *p*-values (above the diagonal) between all pairs of geographic samples (locations) for neutral (a) and outlier (b) data sets and a comparison of the magnitude of those estimates (c). The nine geographic locations are Indian River Lagoon, Florida in the Atlantic (ATL), Tampa Bay, Florida (FLGS), North of Tampa Bay, Florida (FLGN) Mobile Bay, Alabama, (MB), Mississippi Sound, Mississippi (MISS), Chandeleur Sound, LA (CS), off Louisiana west of the Mississippi River (LA), Corpus Christi Bay, Texas (CC), and the Bay of Campeche, Mexico (CAMP). Values significant after correction for multiple testing are bolded and in italics.

a)

| *F*_ST_ -neutral (5,421 loci) | | |  |  |  |  |  |  |  |
| --- | --- | --- | --- | --- | --- | --- | --- | --- | --- |
|  | ATL | FLGS | FLGN | MB | MISS | CS | LA | CC | CAMP |
| ATL | - | ***<0.0001*** | ***<0.0001*** | ***<0.0001*** | ***<0.0001*** | ***<0.0001*** | ***<0.0001*** | ***<0.0001*** | ***<0.0001*** |
| FLGS | ***0.0252*** | - | 0.4823 | ***<0.0001*** | ***<0.0001*** | ***<0.0001*** | ***<0.0001*** | ***<0.0001*** | ***<0.0001*** |
| FLGN | ***0.0246*** | 0.0000 | - | ***<0.0001*** | ***<0.0001*** | ***<0.0001*** | ***<0.0001*** | ***<0.0001*** | ***<0.0001*** |
| MB | ***0.0260*** | ***0.0048*** | ***0.0033*** | - | 0.29027 | 0.58133 | ***0.0029*** | 0.0508 | ***<0.0001*** |
| MISS | ***0.0269*** | ***0.0048*** | ***0.0036*** | 0.0000 | - | 0.2279 | 0.0773 | 0.1122 | ***<0.0001*** |
| CS | ***0.0277*** | ***0.0041*** | ***0.0038*** | 0.0000 | 0.0001 | - | 0.1477 | 0.6684 | ***<0.0001*** |
| LA | ***0.0276*** | ***0.0054*** | ***0.0045*** | ***0.0004*** | 0.0002 | 0.0000 | - | 0.7648 | ***<0.0001*** |
| CC | ***0.0267*** | ***0.0050*** | ***0.0036*** | 0.0002 | 0.0002 | 0.0000 | 0.0000 | - | ***<0.0001*** |
| CAMP | ***0.0479*** | ***0.0248*** | ***0.0237*** | ***0.0225*** | ***0.0225*** | ***0.0218*** | ***0.0224*** | ***0.0202*** | - |

b)

*Fst-non-neutral* (133 loci)

|  | ATL | FLGS | FLGN | MB | MISS | CS | LA | CC | CAMP |
| --- | --- | --- | --- | --- | --- | --- | --- | --- | --- |
| ATL | - | ***<0.0001*** | ***<0.0001*** | ***<0.0001*** | ***<0.0001*** | ***<0.0001*** | ***<0.0001*** | ***<0.0001*** | ***<0.0001*** |
| FLGS | ***0.0867*** | - | ***0.03336*** | ***<0.0001*** | ***<0.0001*** | ***<0.0001*** | ***<0.0001*** | ***<0.0001*** | ***<0.0001*** |
| FLGN | ***0.0916*** | ***0.0023*** | - | ***<0.0001*** | ***<0.0001*** | ***<0.0001*** | ***<0.0001*** | ***<0.0001*** | ***<0.0001*** |
| MB | ***0.1016*** | ***0.0285*** | ***0.0303*** | - | 0.6085 | 0.5284 | 0.2962 | 0.6957 | ***<0.0001*** |
| MISS | ***0.1121*** | ***0.0354*** | ***0.0377*** | 0 | - | 0.6332 | 0.8597 | 0.6204 | ***<0.0001*** |
| CS | ***0.1018*** | ***0.0297*** | ***0.0328*** | 0 | 0 | - | 0.6288 | 0.8881 | ***<0.0001*** |
| LA | ***0.1138*** | ***0.0371*** | ***0.0395*** | 0.0004 | 0 | 0 | - | 0.8287 | ***<0.0001*** |
| CC | ***0.1067*** | ***0.0295*** | ***0.0287*** | 0 | 0 | 0 | 0 | - | ***<0.0001*** |
| Camp | ***0.1351*** | ***0.07065*** | ***0.0680*** | ***0.0919*** | ***0.0939*** | ***0.0906*** | ***0.1022*** | ***0.0845*** | - |

c)

*F*_ST_- *non-neutral F*_ST_/ *F*_ST_ -neutral

|  | ATL | FLGS | FLGN | MB | MISS | CS | LA | CC | CAMP |
| --- | --- | --- | --- | --- | --- | --- | --- | --- | --- |
| ATL |  |  |  |  |  |  |  |  |  |
| FLGS | 3.442 |  |  |  |  |  |  |  |  |
| FLGN | 3.716 | NA |  |  |  |  |  |  |  |
| MB | 3.905 | 5.946 | 9.105 |  |  |  |  |  |  |
| MISS | 4.163 | 7.304 | 10.440 | NA |  |  |  |  |  |
| CS | 3.673 | 7.204 | 8.731 | NA | 0.000 |  |  |  |  |
| LA | 4.121 | 6.827 | 8.791 | 1.100 | 0.000 | NA |  |  |  |
| CC | 3.996 | 5.914 | 8.07 | 0.000 | 0.000 | NA | NA |  |  |
| Camp | 2.821 | 2.8450 | 2.869 | 4.084 | 4.175 | 4.151 | 4.573 | 4.175 |  |

Supplemental Table 3: Pairwise comparisons of gene diversity (*H*_e_) and rarified allelic richness (*A*_r_) for the nine geographic locations using the neutral data set. The nine geographic locations are Indian River Lagoon, Florida in the Atlantic (ATL), Tampa Bay, Florida (FLGS), North of Tampa Bay, Florida (FLGN) Mobile Bay, Alabama, (MB), Mississippi Sound, Mississippi (MISS), Chandeleur Sound, LA (CS), off Louisiana west of the Mississippi River (LA), Corpus Christi Bay, Texas (CC), and the Bay of Campeche, Mexico (CAMP). Significance of pairwise differences was assessed using Wilcoxon signed rank test. The test statistic *Q* is provided below the diagonal and *p*-value above the diagonal, comparisons significant after correction for multiple testing are bolded.

| *He* | ATL | FLGS | FLGN | MB | MISS | CS | LA | CC | CAMP |
| --- | --- | --- | --- | --- | --- | --- | --- | --- | --- |
| ATL | - | **<0.001** | **<0.001** | **<0.001** | **<0.001** | **<0.001** | **<0.001** | **<0.001** | **<0.001** |
| FLGS | **10.596** | - | 0.160 | 0.010 | 0.155 | 0.059 | 0.024 | **0.037** | 0.024 |
| FLGN | **11.472** | 1.406 | - | 0.166 | 0.844 | 0.279 | 0.379 | 0.612 | 0.132 |
| MB | **11.634** | 2.575 | 1.384 | - | 0.151 | 0.742 | 0.244 | 0.502 | 0.514 |
| MISS | **10.365** | 1.423 | -0.196 | 1.436 | - | 0.515 | 0.730 | 0.699 | 0.060 |
| CS | **10.017** | 1.892 | 1.083 | 0.329 | -0.652 | - | 0.648 | 0.657 | 0.189 |
| LA | **10.701** | 2.250 | 0.880 | 1.166 | -0.346 | -0.457 | - | 0.883 | 0.057 |
| CC | **11.075** | **2.083** | 0.507 | 0.671 | 0.387 | -0.445 | 0.147 | - | 0.053 |
| CAMP | **9.337** | 2.264 | 1.506 | 0.652 | 1.879 | 1.314 | 1.900 | 1.934 | - |

| *A_r_* | ATL | FLGS | FLGN | MB | MISS | CS | LA | CC | CAMP |
| --- | --- | --- | --- | --- | --- | --- | --- | --- | --- |
| ATL | **-** | **<0.001** | **<0.001** | **<0.001** | **<0.001** | **<0.001** | **<0.001** | **<0.001** | **<0.001** |
| FLGS | **25.725** | **-** | **0.003** | **<0.001** | **<0.001** | **<0.001** | **<0.001** | **<0.001** | **<0.001** |
| FLGN | **26.142** | **2.971** | **-** | **0.006** | **<0.001** | **<0.001** | **0.033** | **<0.001** | **<0.001** |
| MB | **28.850** | **5.246** | **2.733** | **-** | 0.160 | **0.002** | 0.483 | **0.033** | **<0.001** |
| MISS | **28.018** | **6.190** | **4.516** | -1.407 | **-** | **0.005** | 0.048 | 0.438 | **<0.001** |
| CS | **25.541** | **5.882** | **4.901** | **-3.059** | **-2.838** | **-** | **0.006** | 0.271 | **<0.001** |
| LA | **27.319** | **5.837** | **2.134** | **-**0.701 | 1.981 | **-2.736** | **-** | 0.590 | **<0.001** |
| CC | **28.845** | **7.938** | **4.131** | **-2.137** | 0.776 | -1.102 | -0.539 | **-** | **<0.001** |
| CAMP | **29.932** | **15.490** | **13.370** | **12.431** | **11.370** | **8.345** | **13.440** | **12.244** | **-** |

Supplemental Table 4: Pairwise comparisons of gene diversity (*H*_e_) and rarified allelic richness (*A*_r_) for the nine geographic samples (sites) using the outlier data set. The nine geographic locations are Indian River Lagoon, Florida in the Atlantic (ATL), Tampa Bay, Florida (FLGS), North of Tampa Bay, Florida (FLGN) Mobile Bay, Alabama, (MB), Mississippi Sound, Mississippi (MISS), Chandeleur Sound, LA (CS), off Louisiana west of the Mississippi River (LA), Corpus Christi Bay, Texas (CC), and the Bay of Campeche, Mexico (CAMP). Significance of pairwise differences was assessed using Wilcoxon signed rank test. The test statistic *Q* is provided below the diagonal and *p*-value above the diagonal, comparisons significant after correction for multiple testing are bolded.

| *H_e_* | ATL | FLGS | FLGN | MB | MISS | CS | LA | CC | CAMP |
| --- | --- | --- | --- | --- | --- | --- | --- | --- | --- |
| ATL | - | **0.006** | **0.007** | **0.001** | **0.001** | **<0.001** | **<0.001** | **<0.001** | 0.059 |
| FLGS | **2.771** | - | 0.806 | 0.035 | **0.007** | 0.110 | 0.019 | 0.091 | 0.257 |
| FLGN | **2.718** | 0.246 | - | 0.037 | **0.010** | 0.184 | 0.042 | 0.154 | 0.524 |
| MB | **3.813** | 2.107 | 2.091 | - | 0.055 | 0.572 | 0.667 | 0.115 | 0.052 |
| MISS | **3.983** | **2.699** | **2.574** | 1.922 | - | 0.044 | 0.138 | 0.047 | **0.013** |
| CS | **3.510** | 1.598 | 1.328 | 0.565 | 2.016 | - | 0.341 | 0.802 | 0.075 |
| LA | **3.722** | 2.338 | 2.033 | 0.430 | 1.484 | 0.952 | - | 0.493 | 0.036 |
| CC | **3.504** | 1.690 | 1.424 | 1.575 | 1.990 | 0.251 | 0.686 | - | 0.055 |
| CAMP | 1.882 | 1.135 | 0.637 | 1.940 | **2.477** | 1.781 | 2.100 | 1.921 | - |

| *A_r_* | ATL | FLGS | FLGN | MB | MISS | *CS* | LA | CC | CAMP |
| --- | --- | --- | --- | --- | --- | --- | --- | --- | --- |
| ATL | - | 0.148 | 0.496 | 0.037 | **0.005** | *0.053* | **0.001** | 0.035 | 0.798 |
| FLGS | 1.448 | - | 0.290 | 0.242 | 0.172 | *0.466* | 0.095 | 0.367 | 0.172 |
| FLGN | 0.681 | 1.057 | - | 0.024 | 0.014 | *0.382* | 0.028 | 0.042 | 0.685 |
| MB | 2.085 | 1.169 | 2.265 | - | 0.113 | *0.215* | 0.273 | 0.543 | 0.043 |
| MISS | **2.785** | 1.366 | 2.448 | 1.584 | - | **0.002** | 0.294 | 0.735 | 0.016 |
| CS | 1.936 | 0.729 | 0.874 | 1.241 | **3.147** | *-* | **<0.001** | 0.109 | 0.286 |
| LA | **3.201** | 1.671 | 2.197 | 1.096 | 1.049 | **3.592** | - | 0.451 | **0.002** |
| CC | 2.105 | 0.903 | 2.033 | 0.609 | 0.338 | *1.604* | 0.754 | - | 0.030 |
| CAMP | 0.256 | 1.366 | 0.406 | 2.027 | 2.399 | *1.067* | **3.037** | 2.177 | - |

Supplemental Table 5: Parameter estimates made using Model 3, selected in site frequency spectrum analysis. Long-term effective size estimates (*N*_e_) are shown for a hypothetical ancestral population (Anc) and for each current populations Campeche (CAMP), the northwestern Gulf (nwGulf), the northeastern Gulf (neGulf), and the Atlantic (Atl) currently and a time just post-split (0). Long term migration rates (*m*) are also shown between neighboring populations as are split times (*T*).
